# Short-Term Effects of Swimming Goggle Use on Anterior Segment Parameters in Patients with Keratoconus

**DOI:** 10.3390/medicina62010233

**Published:** 2026-01-22

**Authors:** Nurullah Berk Açar, Atılım Armağan Demirtaş, Tuncay Küsbeci, Mehmet Gencay Çetin

**Affiliations:** 1 Department of Ophthalmology, Izmir City Hospital, 35540 Izmir, Türkiye; drberkacarr@gmail.com (N.B.A.); tkusbeci@yahoo.com (T.K.); mgencaycetin@gmail.com (M.G.Ç.); 2 Department of Ophthalmology, Izmir Faculty of Medicine, University of Health Sciences Turkey, 35540 Izmir, Türkiye

**Keywords:** corneal volume, keratoconus, Scheimpflug imaging, swimming goggles

## Abstract

*Background and Objectives:* Keratoconus is a bilateral but often asymmetric ectatic corneal disease characterized by progressive thinning, increased curvature, and conical shape of the cornea. Previous studies have reported that the use of swimming goggles in patients with keratoconus can lead to increased intraocular pressure (IOP) and a transient reduction in anterior chamber volume (ACV), potentially affecting anterior segment morphology. This study aimed to evaluate the short-term effects of periorbital pressure induced by swimming goggles on corneal parameters in keratoconic eyes. *Materials and Methods:* A total of 44 eyes of 44 patients (mean age: 26.1 ± 5.1 years) diagnosed with keratoconus Stage 1–4 according to the Amsler–Krumeich classification were included. Measurements were taken using a Pentacam^®^ Scheimpflug camera before swimming goggle application and immediately after 20 min of wear. The parameters assessed included keratometry values (K1, K2, Km, Kmax), central and thinnest corneal thickness, corneal volume within the 10 mm zone (CV10), ACV, anterior chamber depth (ACD), iridocorneal angle (ICA), and pupil diameter (PD). *Results*: No statistically significant changes were observed in keratometric values, central and thinnest corneal thickness, ACV, ACD, ICA, PD, or IOP (all *p* > 0.05). CV10 showed a small reduction following goggle wear (Δ = −0.18 mm^3^, corresponding to a 0.3% decrease), which was statistically significant in the unadjusted analysis (*p* = 0.008) but did not remain significant after correction for multiple comparisons (*p* for false discovery rate [FDR] = 0.10). *Conclusions*: Short-term swimming goggle use may induce subtle reductions in CV10 in keratoconic eyes, suggesting a potential biomechanical sensitivity to transient periocular pressure. Although the observed change in CV10 did not retain statistical significance after multiple-comparison correction, it may reflect an early physiological response in structurally compromised corneas. CV measurements could serve as exploratory indicators of mechanical responsiveness in keratoconus, warranting further investigation in larger controlled studies.

## 1. Introduction

Keratoconus (KC) is a bilateral, often asymmetric, ectatic corneal disorder characterized by progressive thinning, increased curvature, and a conical corneal contour. Advances in imaging have improved detection, with prevalence estimates in the general population ranging roughly from 1 in 500 to 1 in 2000, varying by geography [1,2]. Pathogenesis is multifactorial, involving genetic predisposition, inflammatory mediators, oxidative stress, and weakening of stromal collagen architecture [3,4,5]. Disorganization of stromal collagen lamellae together with microdisruptions in Bowman’s layer and Descemet’s membrane compromise biomechanical integrity, making the cornea more susceptible to external loads and facilitating disease progression.

Because of this biomechanical fragility, KC corneas are especially vulnerable to external mechanical forces. Beyond habitual eye rubbing and sleep positions that compress the globe, transient periorbital pressure applied to the ocular surface may also influence corneal biomechanics [6,7,8]. Swimming goggles—which seal to the periocular skin, generate negative periocular pressure, and transiently load the ocular surface—have been reported to produce short-lived increases in intraocular pressure (IOP) and changes in curvature [9,10,11,12,13]. Although the effects of such environmental pressure on a mechanically compromised cornea are not fully delineated, they may resemble some aspects of the mechanical impact of eye rubbing.

Swimming goggles prevent water entry by creating a vacuum, thereby imposing temporary pressure on the periorbital region. In healthy volunteers, several studies have shown mean IOP rises of approximately 2–4 mmHg during wear [10,11,12], and reductions in anterior chamber volume (ACV) have also been reported [14], suggesting acute effects on anterior segment anatomy.

Since KC typically affects younger individuals who often participate in physical or aquatic activities, including swimming, the use of swimming goggles in this population is not uncommon. In such contexts, the use of swimming goggles is necessary, whether for recreational purposes, water sports, or even as protective eyewear in certain occupational or environmental settings. There are a lack of data regarding the effects of swimming goggle use on corneal parameters in patients with KC. While previous studies have mainly focused on IOP fluctuations and anterior chamber changes in healthy eyes, the impact of transient pressure on corneal curvature, thickness, and volume in keratoconic eyes remains unexplored. Due to their compromised biomechanical properties, keratoconic corneas may be more prone to deformation under external compression than normal corneas. Therefore, evaluating the effects of swimming goggle-induced pressure on corneal parameters using Scheimpflug imaging systems may help to fill an important gap in the literature.

The aim of this study was to evaluate the short-term effects of swimming goggle use on corneal parameters in patients with KC using Scheimpflug-based measurements. We set out to determine whether external pressure causes measurable changes in the cornea of KC patients, provide additional information on the importance of mechanical factors in the management of KC, and investigate the mechanisms by which mechanical factors (eye rubbing and sleeping in a prone position) affect corneal biomechanics.

## 2. Materials and Methods

### 2.1. Study Design and Ethics

This prospective, single-center, pre–post observational study adhered to the Declaration of Helsinki and received approval from the Non-Interventional Clinical Research Ethics Committee of Izmir City Hospital (decision date: 23 July 2025, no: 2025/355). All participants received oral and written information about the study and subsequently provided their written informed consent.

### 2.2. Participants

Consecutive patients with KC classified as Stage 1–4 using the Amsler–Krumeich system were enrolled at Izmir City Hospital. Exclusion criteria were corneal scarring, prior ocular surgery (keratoplasty or corneal cross-linking), systemic diseases affecting the cornea, and ocular pathologies other than KC. In total, 44 patients with bilateral KC of a similar stage were included in this study, and the right eyes were selected for analysis in accordance with a predefined protocol to avoid inter-eye correlation and selection bias.

### 2.3. Ophthalmic Examinations and Devices

All participants underwent a comprehensive ophthalmologic examination, including best-corrected visual acuity (BCVA), IOP measurement, and slit-lamp biomicroscopy of the anterior and posterior segments. Objective refraction was obtained using an autorefracto-keratometer (Canon RK-F2, Kawasaki, Japan); BCVA was assessed using a Snellen chart and converted to logarithm of the minimum angle of resolution (logMAR) for the statistical analysis; and IOP was measured using Goldmann applanation tonometry and recorded in millimeters of mercury (mmHg).

Scheimpflug tomography (Pentacam^®^, Oculus, Wetzlar, Germany, software version 1.32r04) was used to obtain anterior segment parameters, including keratometric values (K1, K2, Km, and Kmax in diopters [D]), central corneal thickness (CCT) and thinnest corneal thickness (TCT in micrometers [µm]), corneal volume at the 10 mm zone (CV10 in cubic millimeters [mm^3^]), ACV (mm^3^), anterior chamber depth (ACD in millimeters [mm]), iridocorneal angle (ICA in degrees [°]), and pupil diameter (PD in mm). Corneal tomography measurements were taken in a standardized dark room setting using the three-dimensional anterior chamber analysis module. Participants were instructed to fixate on a central blue light while proper head positioning was ensured. For each eye, only one scan labeled as “OK” by the device’s quality control system was included in the analysis to ensure that the data were reliable.

Axial length (AL) was measured using optical biometry (Nidek AL-Scan, Nidek Co., Gamagori, Japan) and recorded in mm.

All measurements were taken by the same examiner under consistent room illumination. Scans that failed to meet the device’s built-in quality criteria were repeated. To minimize diurnal variation, assessments were scheduled between 13:00 and 16:00. Neither soft nor rigid contact lenses could be worn for at least 1 week before imaging.

### 2.4. Swimming Goggle Fitting

Participants used Aqua Sphere Eagle swimming goggles, which feature a one-piece silicone skirt and an integrated nose bridge frame to ensure a secure and comfortable periorbital seal. For this study, the goggles were equipped with plano (non-prescription) rigid polycarbonate optical lenses. Although the Eagle model supports interchangeable diopter lenses (available in half-step increments from −1.5 to −6.0), no corrective lenses were used in this research. The rigid frame, soft silicone gasket, and integrated nose bridge provide consistent fit and pressure distribution, supporting standardized wear conditions across the participants. To ensure a consistent and symmetrical application of the swimming goggles, each device featured adjustable elastic straps on both sides. Rather than applying a fixed strap length, the tension was customized for each participant by adjusting the strap length so that it was in proportion with the circumference of their head. This method aimed to provide a standardized level of compression across all subjects while accommodating for individual anatomical differences. Before beginning the procedure, the fit and stability of the goggles were carefully checked and modified when needed to maintain uniform application conditions.

### 2.5. Examination Protocol

All measurements were taken on the same day under identical environmental conditions. Prior to goggle wear, each participant underwent a standardized sequence of examinations: corneal tomography, AL measurement, and IOP assessment.

During the 20-min exposure period, the participants remained seated in the waiting area adjacent to the corneal tomography room. Immediately after the goggles were removed, post-exposure measurements were taken within two minutes. Corneal tomography was performed first (approximately 1 min), followed by AL (approximately 1–2 min), and finally IOP measurements (approximately 1–2 min). Both AL and IOP assessments were performed in nearby examination rooms located along the same corridor, minimizing participant movement and potential measurement delays.

### 2.6. Statistical Analysis

The power analysis indicated that a sample size of 44 eyes was required to detect a medium effect size (Cohen’s d = 0.6) with a statistical power of 80% and a significance level of α = 0.05 (E-PICOSAI, MedicRes, New York, NY, USA). Statistical analyses were performed using R software (version 4.4.1; R Core Team, 2024). The normality of the paired differences between before swimming goggle wear (T1) and after swimming goggle wear (T2) measurements were taken was assessed using the Shapiro–Wilk test. For parameters that showed normally distributed differences, paired-sample t-tests were performed, whereas the Wilcoxon signed-rank test was used for non-normally distributed data. Descriptive statistics are presented as mean ± standard deviation (SD) for approximately normally distributed variables and as median with interquartile range (IQR) for non-parametric variables. To assess the clinical relevance of the observed changes, mean differences (Δ = T2 − T1) were reported along with 95% confidence intervals (CIs). Effect sizes were calculated using Cohen’s d for parametric analyses and the Wilcoxon r statistic for non-parametric tests. Since multiple anterior segment parameters were evaluated and no single primary outcome was prespecified prior to study initiation, we applied multiple-comparison correction to control for the increased risk of type I error. To account for this, *p*-values were adjusted using the false discovery rate (FDR) method. An adjusted *p*-value of < 0.05 was considered statistically significant.

## 3. Results

In total, 44 right eyes from 44 KC patients were analyzed. The mean age was 26.1 ± 5.1 years (17–35); 24 participants were female (54.55%) and 20 were male (45.45%). According to the Amsler–Krumeich staging, 15 eyes (34.1%) were Stage 1, 23 (52.3%) were Stage 2, 3 (6.8%) were Stage 3, and 3 (6.8%) were Stage 4. The mean BCVA was 0.12 ± 0.11 logMAR (Table 1).

Before the swimming goggles were worn, the median keratometric values were 47.30 D (IQR: 43.98–49.73) for K1, 50.95 D (IQR: 47.53–52.65) for K2, 48.99 D (IQR: 45.76–50.89) for Km, and 55.40 D (IQR: 51.68–59.92) for Kmax. The median CCT was 471.50 µm (IQR: 447.25–492.25), the TCT was 449.50 µm (IQR: 425.00–481.25), and the median CV10 was 57.90 mm^3^ (IQR: 56.30–59.35).

The anterior chamber metrics included a median ACV of 182.10 mm^3^ (IQR: 165.60–202.00) and a median ACD of 3.22 mm (IQR: 3.06–3.45). The median ICA was 40.10° (IQR: 36.45–43.75), and the PD was 3.30 mm (IQR: 2.85–3.85) (Table 2).

**Figure 1:**
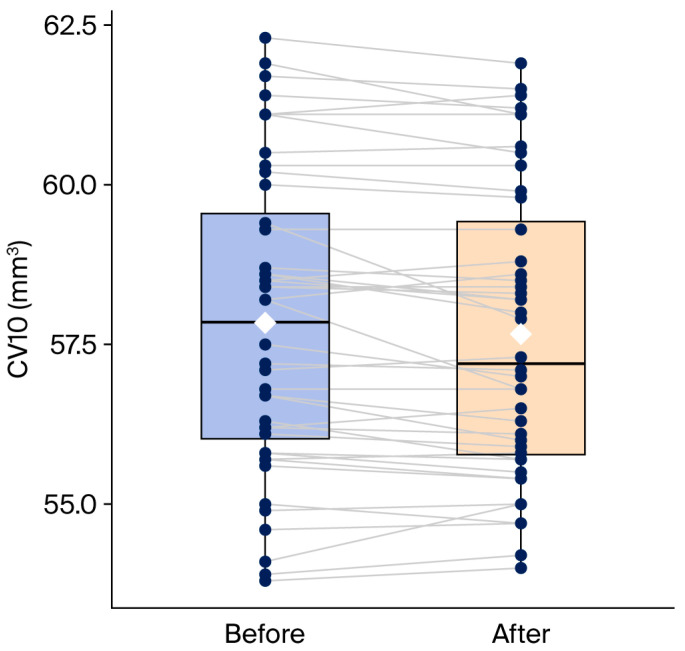
The change in corneal volume in 10 mm zone (CV10) before and after swimming goggle wear. Black dots represent the individual CV10 measurement at the corresponding time points connected by paired grey lines. The white squares indicate the mean CV10 values for each condition.

Following 20 min of swimming goggle wear, none of the keratometric parameters (K1, K2, Km, Kmax) demonstrated a statistically significant change (all *p* > 0.05). Similarly, the changes in CCT and TCT were not significant (*p* = 0.606 and *p* = 0.386, respectively). CV10 showed a small reduction from 57.83 ± 2.36 mm^3^ to 57.66 ± 2.26 mm^3^ (Δ = −0.18 mm^3^, corresponding to a 0.3% decrease). The change in CV10 before and after swimming goggles were worn is presented in Figure 1. While this decrease reached statistical significance in the unadjusted analysis (*p* = 0.008), it did not remain significant after correction for multiple comparisons using the FDR method (pFDR = 0.10).

Other anterior segment parameters, including ACD, ACV, ICA, and PD, did not show significant differences between the pre- and post-wear measurements (all *p* > 0.05; all pFDR > 0.05). IOP exhibited a slight, non-significant increase, and the AL measurements remained stable after goggle wear (Table 2).

## 4. Discussion

Following short-term swimming goggle wear, no statistically significant changes were observed in any of the evaluated anterior segment parameters after adjusting for multiple comparisons. While a slight reduction in CV10 was initially significant in the raw analysis, this finding did not remain statistically significant after controlling for multiplicity. These results suggest that short-term periocular pressure from swimming goggles does not induce clinically meaningful or robust biomechanical changes in keratoconic corneas.

The mechanical stress induced by swimming goggles has been hypothesized to produce biomechanical effects that resemble those caused by chronic eye rubbing, a well-established risk factor in the pathogenesis and progression of KC. McMonnies suggested that repeated eye rubbing may contribute to corneal weakening through mechanisms such as transient IOP elevation (reportedly exceeding 150 mmHg), slippage of collagen lamellae, epithelial thinning, and enzymatic degradation of the stromal matrix [9]. Although the mechanical stimulus in our study was brief, standardized, and considerably less intense than habitual eye rubbing, a slight reduction in CV10 was observed. While this change did not remain statistically significant after correction for multiple comparisons, its directionality may still reflect a mild, transient biomechanical response to external compression. Notably, no significant change in IOP was recorded in our cohort, suggesting that the short-duration pressure applied by the goggles was insufficient to induce measurable hydrostatic effects in the anterior chamber. Nevertheless, these subtle findings support the notion that keratoconic corneas may exhibit heightened mechanical sensitivity, even to transient periocular pressure.

Beyond purely mechanical pathways, short episodes of stress may also trigger inflammatory changes. Balasubramanian et al. showed that just one minute of eye rubbing in healthy subjects elevated proinflammatory tear-film markers [15]. Since we observed a short-term volume change, further investigation into combined mechanical–biochemical responses in individuals with KC is necessary.

Ulusoy and Duru assessed the effects of short-term goggle wear in individuals with KC (74 eyes of 37 patients; mean age 25.97 ± 7.7 years; mean Kmax 52.72 ± 5.36 D), with measurements taken at 1, 10, and 20 min. They reported no changes in keratometry, corneal thickness, or CV, but they did find a significant decrease in ACV (*p* < 0.001) [14]. Our study included 44 patients (mean age 26.1 ± 5.1 years) across a broader severity spectrum and a higher mean Kmax (56.30 ± 6.48 D), with 52.3% at Stage 2. In contrast to Ulusoy and Duru’s study, where staging data and Km values were not reported, our cohort’s distribution was characterized by explicit staging and higher Kmax values, allowing for a more nuanced interpretation of disease severity. We analyzed a single eye per subject to avoid inter-eye dependency. In our study, no statistically significant changes were observed in any of the evaluated tomographic parameters, including keratometry, corneal thickness, ACV, or ACD. While CV10 initially showed a statistically significant reduction after 20 min of goggle wear, this finding did not remain significant after correction for multiple comparisons. The differences between these studies may relate to goggle design, stage distribution, or methodological nuances.

In healthy young adults, Bilici et al. reported transient reductions in CCT, ACD, and ICA after goggle wear, with rapid return to baseline following removal [16]. However, they removed the goggle’s front lens during the measurement, compromising the negative-pressure environment and thus real-world fidelity. Jiménez et al. used a perforated front lens and observed decreases in CCT and ICA with an average 4 mmHg IOP increase during wear, which again normalized after removal; ACD and ACV were unchanged [11]. Zhang et al. compared an eye-socket design with a wide-frame orbital design in healthy adults; for measurement convenience, the right-eye front lens was removed in both designs. During wear, IOP rose and the anterior chamber parameters fell, returning to baseline upon removal [12]. In all such setups, removing or perforating the front lens compromises the integrity of the negative pressure, limiting generalizability to real-world use; further, findings in healthy eyes may not translate to biomechanically fragile KC corneas.

Prior studies have consistently demonstrated that total CV decreases with disease severity and may serve as a morphometric indicator of ectatic progression [17,18,19,20,21,22]. Ambrósio et al. [19] and Mannion et al. [20] emphasized this structural depletion, while Çağıl et al. [21] and Wu et al. [22] further established the diagnostic relevance of CV—particularly within the 3 mm central zone—as a highly sensitive metric for early KC detection.

One potential explanation for the lack of a statistically significant change in CV10 after correction for multiple comparisons may lie in the limitation of using only global corneal volume (10 mm zone) for the analysis. Since KC typically affects the central and paracentral cornea more prominently, subtle yet clinically relevant alterations may have occurred in smaller zones that were not captured by a global CV metric. For instance, Çağıl et al. reported that CV in the 3 mm zone was 3.80 mm^3^ in normal eyes, 3.56 mm^3^ in subclinical KC eyes, and 3.38 mm^3^ in clinical KC eyes, reflecting a more pronounced percentage difference (~5.8%) than in CV10 (~6.1% from normal to KC, but only ~0.7% between subclinical and clinical KC). These findings suggest that regional volume measurements—particularly in the central 3 mm zone—may be more sensitive to detecting early or subtle biomechanical responses. Although our observed CV10 reduction is numerically smaller, it occurred in response to a short-term mechanical stressor rather than pathological progression, emphasizing the cornea’s biomechanical sensitivity. Therefore, even a 0.3% decrease may reflect early mechanical responsiveness, especially in structurally compromised keratoconic corneas, supporting the potential utility of CV metrics as dynamic indicators in assessing environmental susceptibility in such patients.

The reduction in CV10—despite not meeting the threshold for statistical significance after correction for multiple comparisons—was not accompanied by a change in CCT following short-term goggle wear. This discrepancy may indicate a transient redistribution of stromal fluid rather than an actual loss of CV. Previous studies have shown that external mechanical forces—such as eye rubbing or contact lens wear—can cause regional fluctuations in corneal hydration without uniformly affecting all areas of the cornea [23,24]. Considering that swimming goggles exert circumferential periocular pressure, it is plausible that fluid shifts occur from the central cornea toward the periphery. This mechanism may lead to a measurable decrease in global CV while maintaining CCT. Similar biomechanical responses have been described in early KC and under mechanical stress, where the central and peripheral stroma respond differently depending on the gradient of corneal stiffness and hydration [25]. These findings support the hypothesis that localized pressure may preferentially influence the peripheral cornea, resulting in subtle yet detectable changes in volume distribution.

Our findings should be interpreted in the context of previous observations made by Cavas-Martínez et al., who emphasized that global CV measurements, while statistically meaningful, may lack the sensitivity to detect subtle and localized biomechanical changes in the early stages of KC. They highlighted the diagnostic value of zone-specific analyses, particularly in regions where the cone tends to develop [26]. Although we recognize this methodological nuance, our study was limited to CV10, the only zone for which data were consistently available due to software constraints. As such, zone-specific CV values (e.g., 3 mm, 5 mm, 7 mm) could not be analyzed.

Despite this situation, our study bridges a relevant gap between structural tomographic metrics and real-world external mechanical stress. CV10 may still serve as a dynamic indicator of corneal responsiveness in biomechanically vulnerable eyes, and its post-wear reduction suggests potential sensitivity to environmental triggers. These findings underscore the importance of individualized guidance for patients with KC, including being cautious when considering the use of periocular devices such as swimming goggles. Further studies incorporating zone-specific volume metrics are warranted to better capture regional susceptibility and early biomechanical compromise in individuals with KC.

One of the strengths of our study is the use of standard, unmodified swimming goggles, which closely simulates real-world usage conditions. Unlike some prior studies where goggle lenses were perforated or removed, our approach preserved the periorbital negative-pressure environment, thereby more accurately reflecting the physiological effects of goggle wear. We also employed high-resolution Pentacam^®^ Scheimpflug tomography for the measurements, allowing for robust and sensitive assessments of parameters like CV to be provided. Moreover, while most studies focused on the ocular effects of swimming goggles have been conducted in healthy individuals, few have addressed this issue in KC patients. Our study fills this research gap by evaluating the short-term impact of goggle wear in KC patients with methodological rigor, offering a unique contribution to the literature. In addition, unlike many previous studies, we applied rigorous statistical methods—including effect size reporting and correction for multiple comparisons—to ensure that our findings were both robust and interpretable.

Nonetheless, our study has several limitations. The sample size was modest and stage distribution was heterogeneous, limiting subgroup analyses. Since the majority of eyes in our cohort were classified as Stage 1 or 2 (86%), our findings may primarily reflect early-to-moderate KC and may not fully capture biomechanical responses in more advanced stages, where stromal thinning and structural compromise are more pronounced. Only one goggle model was tested, meaning that conclusions about different designs or pressure profiles cannot be made. Wear duration was limited to 20 min; longer use and underwater pressure were not evaluated. Measurements were taken before wear and shortly after removal; it was not possible to perform in situ assessments during wear due to equipment constraints. Although only short-term effects were assessed, cumulative or repetitive goggle wear may lead to greater biomechanical alterations, especially in individuals with advanced KC. Furthermore, only CV10 was available for all participants; due to software limitations, zone-specific CV values (e.g., 3 mm, 5 mm, 7 mm) could not be analyzed. In particular, the absence of zone-specific CV data limited our ability to evaluate whether regional CV changes were more pronounced in specific zones, which may be especially relevant in keratoconic corneas where structural alterations tend to localize. This remains an important area for future studies utilizing high-resolution topographic analysis. We also acknowledge the importance of localized CV analysis and plan to include full zonal data in future investigations once full software access becomes available. Additionally, this study did not include a healthy control group, which limits our ability to make comparisons; future studies should incorporate age- and sex-matched controls to better contextualize the biomechanical responsiveness observed in keratoconic eyes. Finally, we did not include deformation-based biomechanical testing, focusing solely on tomographic parameters. Future work should include larger cohorts, different goggle designs, variable wear durations/pressures, and, where feasible, in situ imaging. Experimental or computational models simulating underwater pressure on KC corneas may yield additional insights.

## 5. Conclusions

In conclusion, this study is among the few to assess the short-term effects of wearing swimming goggles on corneal parameters in individuals with KC. Following 20 min of use, no statistically significant changes were observed in keratometric values, corneal thickness, anterior chamber metrics, or pupil diameter after adjustment for multiple comparisons. Although a slight decrease in CV10 was initially significant, this finding did not remain significant after correction for multiple testing, suggesting that the observed change may reflect a subtle and transient biomechanical response rather than a definitive morphological alteration.

Nonetheless, given the known biomechanical fragility of the keratoconic cornea, even brief periods of periocular pressure—such as that exerted by swimming goggles—may influence stromal behavior. This highlights the importance of considering the mechanical interaction of periocular devices in KC patients. Thus, when selecting swimming goggles for individuals with KC, clinicians should consider not only optical correction but also mechanical interaction with the ocular surface. While no major clinical impact was detected in our cohort, the potential sensitivity of CV metrics to mechanical stimuli suggests that they may serve as useful markers in future studies investigating environmental influences on disease progression. Further research with healthy controls, longer exposure times, and a variety of goggle designs is warranted to better understand the clinical implications of such transient stressors.

## Figures and Tables

**Table 1:** Demographic features of patients and clinical characteristics of eyes (n = 44).

Parameters	Value (Mean ± SD or n, %)
Age (years)	26.1 ± 5.1
Sex (female/male)	24 (54.55%)/20 (45.45%)
Best-corrected visual acuity (logMAR)	0.12 ± 0.11
Keratoconus staging (Amsler–Krumeich)	
Stage 1	15 (34.1%)
Stage 2	23 (52.3%)
Stage 3	3 (6.8%)
Stage 4	3 (6.8%)

SD = standard deviation; logMAR = logarithm of the minimum angle of resolution.

**Table 2:** Comparison of parameters before and after swimming goggle wear.

Parameters	Time	Mean (SD)/Median (IQR)	Change (Δ)/%95 CI	Effect Size	*p*	*p* (FDR)
K1 (D)	T1	46.78 ± 4.25/46 [44.6–48.37]	0.38 ± 2.71/0 [−0.2–0.2]	0.01 ^b^	0.916	0.916
	T2	47.17 ± 4.21/46.2 [45–48.52]	[−0.1, 0.1]			
K2 (D)	T1	50.34 ± 4.32/49.7 [47.98–52.08]	0.11 ± 1.65/0 [−0.3–0.1]	0.17 ^b^	0.236	0.413
	T2	50.45 ± 4.46/49.8 [47.85–52.02]	[−0.25, 0.1]			
Km (D)	T1	48.48 ± 4.19/47.8 [46.18–49.8]	0.28 ± 2.26/0 [−0.12–0.1]	0.164 ^b^	0.286	0.413
	T2	48.75 ± 4.28/47.9 [46.53–50.6]	[−0.15, 0.05]			
Kmax (D)	T1	56.31 ± 6.48/55.55 [53.22–59.85]	0.04 ± 1.81/−0.05 [−0.43–0.23]	0.137 ^b^	0.35	0.413
	T2	56.35 ± 6.49/55.7 [53.32–59.95]	[−0.35, 0.15]			
CCT (µm)	T1	471.91 ± 40.51/477.5 [445.5–500]	−1.02 ± 13.04/1 [−2–3.25]	0.173 ^b^	0.327	0.413
	T2	470.89 ± 41.94/478 [439.75–496]	[−1, 2.5]			
TCT (µm)	T1	452.09 ± 45.49/455.5 [416.25–478]	0.89 ± 6.71/3 [−1.25–5]	0.291 ^b^	0.065	0.388
	T2	452.98 ± 45.76/453.5 [422.75–480]	[0, 3.5]			
CV10 (mm^3^)	T1	57.84 ± 2.36/57.85 [56.03–59.55]	−0.18 ± 0.43/−0.15 [−0.33–0.1]	0.399 ^b^	0.008	0.1
	T2	57.66 ± 2.27/57.2 [55.77–59.42]	[−0.3, −0.05]			
ACV (mm^3^)	T1	181.11 ± 33.57/178 [158–208]	−0.77 ± 4.39/−1 [−3–2]	0.176 ^a^	0.249	0.413
	T2	180.34 ± 33.8/175.5 [156.75–205.5]	[−2.11, 0.56]			
ACD (mm)	T1	3.23 ± 0.31/3.3 [2.96–3.48]	0 ± 0.07/0 [−0.03–0.01]	0.171 ^a^	0.31	0.413
	T2	3.23 ± 0.33/3.33 [2.96–3.48]	[−0.02, 0.01]			
ICA (°)	T1	40.1 ± 5.32/40.1 [36.98–42.9]	0.78 ± 2.98/0.6 [−0.65–1.8]	0.262 ^a^	0.09	0.388
	T2	40.88 ± 5.42/41.4 [37.85–44.37]	[−0.13, 1.68]			
PD (mm)	T1	3.43 ± 0.63/3.42 [2.87–3.79]	−0.08 ± 0.42/−0.06 [−0.18–0.08]	0.223 ^b^	0.149	0.413
	T2	3.34 ± 0.48/3.33 [2.95–3.67]	[−0.15, 0.02]			
IOP (mmHg)	T1	13.27 ± 2.72/13 [11–15]	0.07 ± 1.15/0 [−1–1]	0.052 ^b^	0.775	0.839
	T2	13.34 ± 2.58/13 [11.75–14.25]	[−0.5, 1]			
AL (mm)	T1	24.14 ± 1.06/24.2 [23.4–24.98]	0.01 ± 0.17/0.02 [−0.09–0.11]	0.145 ^b^	0.342	0.413
	T2	24.15 ± 1.04/24.22 [23.51–24.98]	[−0.02, 0.07]			

SD = standard deviation; IQR = interquartile range; CI = confidence interval; FDR = false discovery rate; D = diopter; T1 = before swimming goggle wear; T2 = after swimming goggle wear; CCT = central corneal thickness; TCT = thinnest corneal thickness; CV10 = corneal volume within the 10 mm zone; ACV = anterior chamber volume; ACD = anterior chamber depth; ICA = iridocorneal angle; PD = pupil diameter; IOP = intraocular pressure; AL = axial length. ^a^: Paired-sample *t*-test (Cohen’s d). ^b^: Wilcoxon signed-rank test (Wilcoxon r). *p* (FDR) < 0.05 was considered statistically significant.

## Data Availability

The data presented in this study are available on request from the corresponding author due to ethical reasons.
